# Первый опыт применения ингибитора ароматазы в сочетании с гормоном роста с целью улучшения ростового прогноза у подростка 14 лет с дефицитом гормона роста после лечения краниофарингиомы. Описание клинического случая

**DOI:** 10.14341/probl13446

**Published:** 2024-05-21

**Authors:** Н. А. Мазеркина, С. К. Горелышев, А. Н. Саватеев, Н. А. Стребкова, А. Л. Калинин

**Affiliations:** Центр нейрохирургии им. акад. Н.Н. Бурденко; Центр нейрохирургии им. акад. Н.Н. Бурденко; АО «Деловой центр нейрохирургии». Центр «Гамма-нож»; Научный медицинский исследовательский центр эндокринологии; Научный медицинский исследовательский центр эндокринологии

**Keywords:** краниофарингиома, гормон роста, дефицит гормона роста, ингибитор ароматазы

## Abstract

В статье описывается первый опыт успешного применения гормона роста (ГР) в сочетании с ингибитором ароматазы (ИА) у мальчика 14 лет. В возрасте 7 лет у него появились головные боли с тошнотой и рвотой, на МРТ была выявлена краниофарингиома (КФ). Была установлена система Оммайя и проведена лучевая терапия. В результате лечения развился дефицит гормона роста (ГР) и вторичный гипотиреоз. В 9 лет появились признаки полового созревания. Темпы роста до 14 лет оставались удовлетворительными. В 14 лет скорость роста замедлилась, что послужило причиной обращения. При обследовании костный возраст составил 16 лет, ожидаемый конечный рост без терапии — 162 см. Учитывая плохой ростовой прогноз, был назначен ИА анастрозол в сочетании с ГР. В течение двух лет терапии прибавка в росте составила 12,5 см. Данное наблюдение показывает, что нормальные темпы роста у больных с КФ не свидетельствуют о сохранной соматотропной функции гипофиза. При сохранной половой функции может наблюдаться раннее или преждевременное половое созревание. В таких случаях, помимо ГР, могут назначаться ИА — препараты, тормозящие закрытие зон роста. Терапия ГР в сочетании с ИА высокоэффективна и безопасна у пациентов с дефицитом ГР после лечения КФ в период полового созревания и позволяет достичь хороших ростовых показателей.

## Abstract (en)

This article describes the first experience of successful use of growth hormone (GH) in combination with an aromatase inhibitor (AI), in a 14-year-old boy. At the age of 7, he presented with headaches, nausea and vomiting, and MRI revealed a craniopharyngioma (CP). An Ommaya system was implanted, and radiation therapy was performed. As a result of treatment, GH deficiency and secondary hypothyroidism developed. At age 9 years, signs of puberty appeared. Growth rate remained satisfactory until the age of 14 years. At the age of 14 growth rate slowed down, which was the reason for appointment. Upon examination, the bone age was 16 years and the projected final height without therapy was 162 cm. Given the poor growth prognosis, IA anastrozole in combination with GH was prescribed. During two years of therapy the growth gain amounted to 12.5 cm. This observation demonstrates that normal growth rates in patients with CP do not indicate preserved somatotropic function of the pituitary gland. With preserved sexual function, early or premature puberty may be observed. In such cases, IA can be prescribed in addition to GH — these are medications that inhibit the closure of growth. GH therapy in combination with IA is highly effective and safe in patients with GH deficiency after treatment of KF during puberty and allows to achieve good growth parameters.

## АКТУАЛЬНОСТЬ

Рост у детей остается важным показателем здоровья и социальной адаптации. После лечения опухолей мозга может наблюдаться задержка роста, обусловленная дефицитом ГР и нарушением полового созревания. Своевременная диагностика таких состояний и назначение адекватной терапии позволяют достичь удовлетворительных показателей конечного роста.

## ОПИСАНИЕ СЛУЧАЯ

В возрасте 7 лет у мальчика появились головные боли с тошнотой и рвотой. Он был обследован, при МРТ выявлена краниофарингиома (КФ) (рис. 1а). Была проведена операция — биопсия опухоли и установка системы Оммайя с эвакуацией кистозного содержимого с последующей протонотерапией. После лечения развился вторичный гипотиреоз, по поводу которого назначена заместительная терапия препаратом левотироксина.

В дальнейшем пациент находился под наблюдением нейрохирурга (рецидива опухоли не было, рис. 1б) и эндокринолога по месту жительства. Исследование функции ГР не проводилось в связи с нормальными темпами роста (рис. 2). В 14 лет рост замедлился до 2 см в год, что послужило причиной обращения к эндокринологу в НМИЦ нейрохирургии им. акад. Н.Н. Бурденко.

**Figure:**
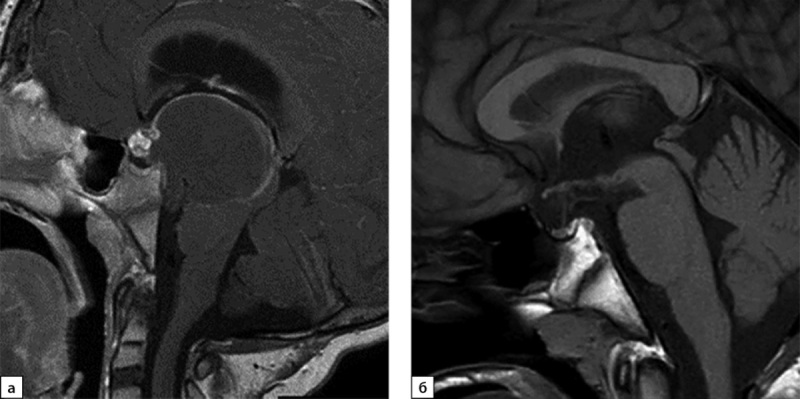
Рисунок 1. МРТ пациента с КФ (а — до операции, б — на момент обращения).

**Figure:**
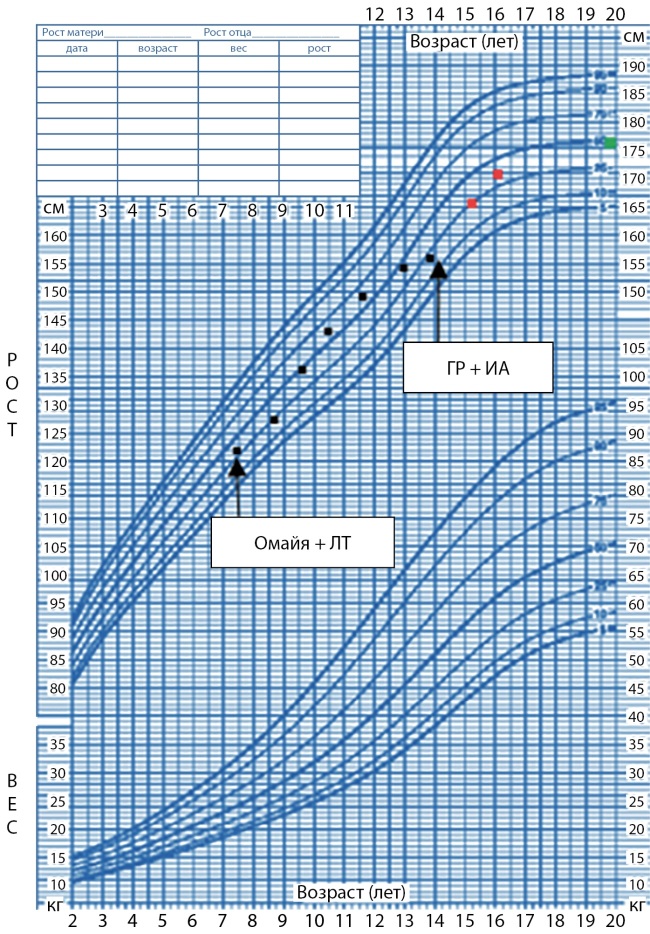
Рисунок 2. Ростовая кривая пациента до и после назначения ИА.

## Результаты физикального, лабораторного и инструментального обследований

При осмотре в 14,0 лет: рост — 157,5 см (SDS роста=-0,82), рост родителей: мама — 165 см, папа — 175 см, целевой рост — 176,5 см. Получает Л-тироксин 75 мкг в сутки. Половое развитие по Таннеру — 4, объем тестикул — 15 мл. Со слов, первые симптомы полового созревания появились в 9 лет. Костный возраст — 16 лет (рис. 3). В анализе крови на гормоны (табл. 1) все показатели в пределах нормы, кроме уровня инсулиноподобного фактора роста 1 (ИФР-1) — 130 нг/мл (SDS ИФР-1 составил -2,3). Ожидаемый конечный рост без терапии — 162 см.

**Figure:**
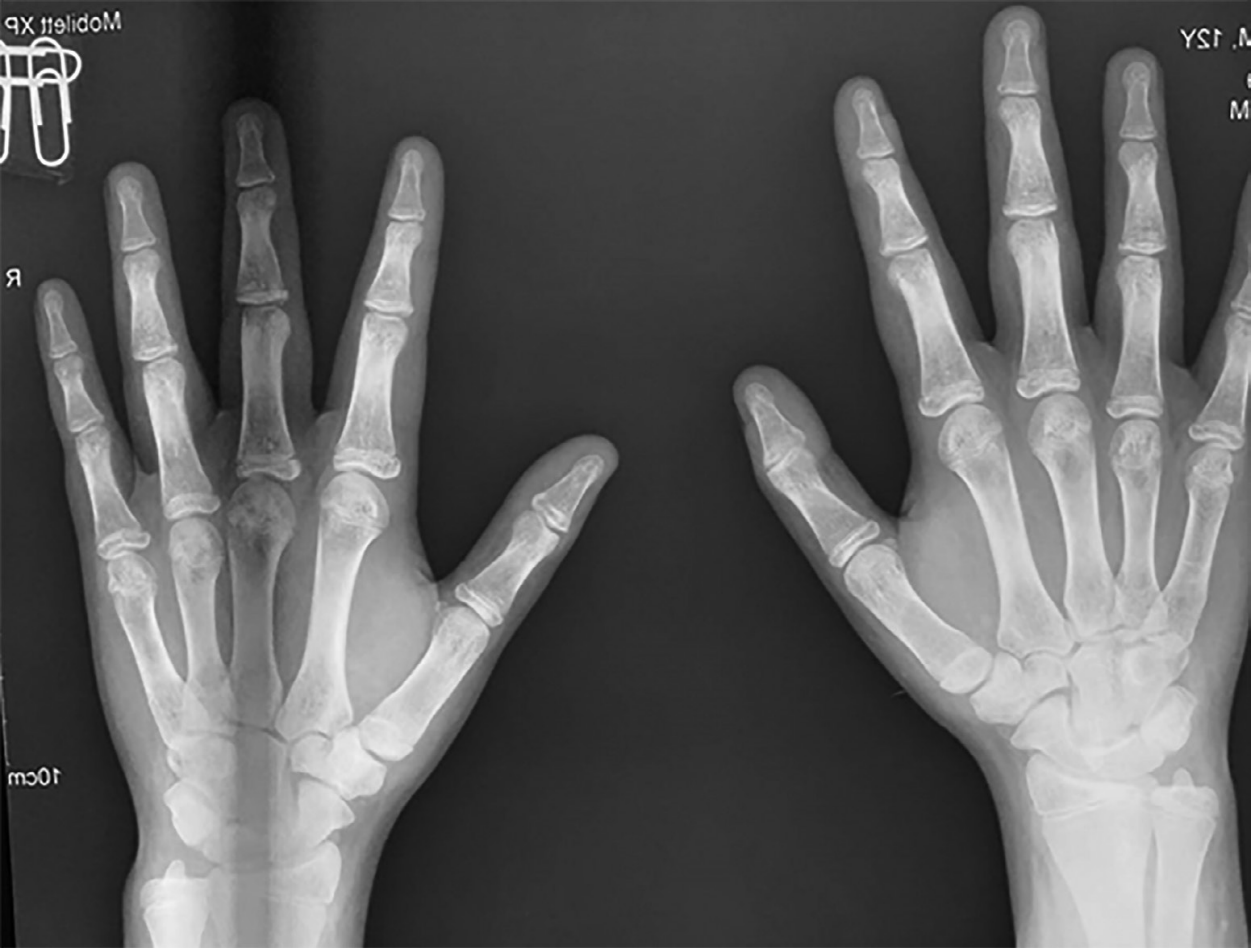
Рисунок 3. Костный возраст (16 лет) пациента до терапии.

**Table:** Таблица 1. Уровни показателей крови у пациента до и на фоне назначения ГР с ИА

Показатели крови	До назначения ГР и ИА	На фоне приема ГР и ИА	Норма
ТТГ, мкЕд/л	0,01	0,03	0,4–4,0
свТ4, пМоль/л	15,6	16,5	10,7–18,4
кортизол, нМоль/л	385	512	101–536
пролактин, мЕд/л	132	214	85–328
ЛГ, мЕд/л	3,0	10	0–7
ФСГ, мЕд/л	3,45	4,3	1,4–7,5
тестостерон, нМоль/л	28,0	36	0,31–18,5
ИФР-1, нг/мл	130	583	177–607
гемоглобин, г/л	128	175	120–160

## Лечение

Учитывая неудовлетворительный ростовой прогноз, наличие дефицита ГР в исходе лечения КФ, пациенту была назначена заместительная терапия ГР в дозе 0,033 мг на кг массы тела в сутки в сочетании с ИА с целью замедления костного созревания анастрозолом 1 мг в сутки. Проводился контроль гормональных показателей (табл. 1), биохимического и общего анализов крови. Из побочных эффектов отмечалось появление угревой сыпи на лице, а также повышение уровня гемоглобина до 175 г/л (табл. 1) при гематокрите 56%, в дальнейшем на фоне терапии уровень гемоглобина нормализовался.

## Исход и результаты последующего наблюдения

Данную терапию пациент получал в течение двух лет, на этом фоне вырос со 157,5 до 170 см (на 12,5 см) (рис. 2). В настоящее время пациент продолжает получать терапию, данных о его конечном росте пока нет.

## ОБСУЖДЕНИЕ ПОЛУЧЕННЫХ РЕЗУЛЬТАТОВ

КФ — доброкачественные опухоли, удаление которых приводит к пангипопитуитаризму у 61–100% пациентов [1][2]. В описанном нами случае опухоль имела преимущественно кистозное строение, в связи с чем ее удаление не проводилось, стереотаксически была установлена система Оммайя с последующей эвакуацией кистозного содержимого и протонотерапией. Облучение после операции также вызывает дефицит гормонов гипофиза. Наиболее чувствительной к повреждающему воздействию является функция ГР [3]. Учитывая наличие у пациента гипотиреоза, сопутствующий дефицит ГР не вызывал сомнений, в связи с чем стимуляционные тесты для исследования функции ГР пациенту не проводились. ИФР-1 отражает суммарную секрецию ГР, его нормативные значения зависят от возраста и стадии полового созревания [4]. В данном клиническом случае сниженный уровень ИФР-1 также свидетельствовал о дефиците ГР у пациента.

Безопасность терапии ГР у пациентов с различными опухолями мозга, в частности КФ, остается предметом дискуссии, хотя на сегодняшний день не выявлено увеличения частоты рецидивов на фоне лечения [5]. Так, в наиболее крупной международной базе данных KIGS, куда включен 1381 пациент с КФ, частота рецидивов составила 11,9%, что не выше, чем у пациентов, не получавших ГР [6].

Несмотря на дефицит ГР, часть больных может хорошо расти за счет других факторов, а именно за счет повышенной биодоступности ИФР-1 [1], но с течением времени скорость роста обычно снижается. Поэтому оценка функции ГР и ростового прогноза с назначением при необходимости заместительной терапии должна проводиться у всех пациентов с КФ независимо от темпов роста.

Критическим периодом, когда исчерпывается эффективность рост-стимулирующего действия ГР ввиду закрытия зон роста под воздействием половых стероидов, является период полового созревания. После лечения КФ у большинства пациентов отмечается сопутствующий гипогонадизм. В этих случаях заместительную терапию половыми гормонами можно начинать в любом возрасте, когда рост пациента достиг удовлетворительных показателей. У пациентов с сохранной половой функцией рост-стимулирующая терапия ГР ограничена по времени.

Одним из последствий облучения при различных опухолях мозга может быть раннее или преждевременное половое созревание (в том числе и при КФ, если сохранна половая функция), пубертат в среднем наступает на 2 года раньше, чем в общей популяции [7]. Скорость роста в таких случаях может быть нормальной, но пациенты не реализуют свой ростовой скачок в период полового созревания, и вследствие этого конечный рост может быть низким, особенно у пациентов с сопутствующим дефицитом ГР, как в описанном нами случае.

С целью замедления костного созревания в таких случаях возможно назначение аналогов гонадолиберина, однако это приводит к развитию гипогонадизма [8].

В различных исследованиях было показано, что среди половых гормонов именно эстрогены оказывают основное влияние на костное созревание [9]. Эстрогены уменьшают количество клеток — предшественников хондроцитов в фазе покоя, способствуя стабильности структуры хряща [10]. Таким образом блокада эстрогенов может задерживать костное созревание, не вызывая гипогонадизма у подростков мужского пола [11]. Эстрогены синтезируются из тестостерона путем ароматизации с помощью фермента ароматазы. Поэтому назначение ИА блокирует синтез эстрогенов в организме, и таким образом замедляется закрытие зон роста.

На фоне терапии по принципам обратной связи может повышаться секреция ЛГ и тестостерона, что мы и видели у нашего пациента. Повышение уровня тестостерона привело к появлению угревой сыпи и повышению уровня гемоглобина [12]. Данная терапия позволила увеличить скорость роста пациента с 2 см в год до 6 см в год, в целом за два года лечения прибавка в росте составила 12,5 см.

В литературе данные об эффективности терапии БА достаточно разнородны. В недавнем кохрановском обзоре, анализировавшем 4 исследования в совокупности 84 пациентов с различными формами низкорослости (дефицит ГР, идиопатическая низкорослость, конституциональная задержка роста и пубертата), несмотря на то, что прогнозируемый конечный рост увеличился на 5,1–6,1 см во всех исследованиях по сравнению с контрольной группой, улучшений конечного роста не было [13]. Правда, конечный рост анализировался в двух исследованиях из четырех. В одном из последних метаанализов, анализирующих эффективность БА и аналогов гонадолиберина в монотерапии у пациентов с идиопатической низкорослостью, прибавка конечного роста на фоне БА составила 4,9 см [14]. У нашего пациента конечный рост улучшился со 162 до 170 см, такой хороший эффект обусловлен, вероятно, комбинированной терапией ГР и БА у пациента с соматотропной недостаточностью.

В опубликованной на сегодняшний день литературе нет сообщений о применении ИА в сочетании с ГР у детей с опухолями мозга, в том числе при КФ.

В настоящее время мальчик продолжает получать терапию ГР и ИА, данных о его конечном росте пока нет.

## ЗАКЛЮЧЕНИЕ

Нормальные темпы роста у пациентов с КФ не свидетельствуют о сохранности функции ГР. При сохранной половой функции может наблюдаться раннее или преждевременное половое созревание. В таких случаях, помимо ГР, могут назначаться ИА — препараты, тормозящие закрытие зон роста. Терапия ГР в сочетании с ИА высокоэффективна и безопасна у пациентов с дефицитом ГР после лечения КФ в период полового созревания и позволяет достичь хороших ростовых показателей.

## СПИСОК СОКРАЩЕНИЙ

ГР — гормон роста

КФ — краниофарингиома

ИА — ингибиторы ароматазы

ИФР-1 — инсулиноподобный фактор роста 1

## ДОПОЛНИТЕЛЬНАЯ ИНФОРМАЦИЯ

Источники финансирования. Работа выполнена по инициативе авторов без привлечения финансирования.

Конфликт интересов. Авторы декларируют отсутствие явных и потенциальных конфликтов интересов, связанных с содержанием настоящей статьи.

Участие авторов. Мазеркина Н.А. — сбор и обработка материала, интерпретация результатов, написание текста, подготовка рукописи; Горелышев С.К. — критическая интерпретация результатов, внесение существенной правки, одобрение финальной версии; Саватеев А.Н. — подготовка рукописи; Калинин А.Л. — интерпретация результатов, подготовка рукописи, внесение существенной правки; Стребкова Н.А. — интерпретация результатов, подготовка рукописи, внесение существенной правки.

Все авторы одобрили финальную версию статьи перед публикацией, выразили согласие нести ответственность за все аспекты работы, подразумевающую надлежащее изучение и решение вопросов, связанных с точностью или добросовестностью любой части работы.

Согласие пациента. Законный представитель пациента добровольно подписал информированное согласие на публикацию персональной медицинской информации в обезличенной форме в этом журнале.
